# Successful pregnancy after in vitro fertilization in an ABO-incompatible kidney transplant recipient receiving rituximab: a case report

**DOI:** 10.1186/s12882-019-1396-9

**Published:** 2019-06-06

**Authors:** Akihiro Kosoku, Junji Uchida, Keiko Maeda, Yuki Yoshikawa, Akihiro Hamuro, Hisao Shimada, Kazuya Kabei, Shunji Nishide, Tomoaki Iwai, Nobuyuki Kuwabara, Toshihide Naganuma, Norihiko Kumada, Yoshiaki Takemoto, Tatsuya Nakatani

**Affiliations:** 1 Department of Urology, Osaka City University Graduate School of Medicine, 1-4-3, Asahi-machi, Abeno-ku, Osaka, 545-8585 Japan; 2 Department of Nursing, Osaka City University Hospital, 1-5-7, Asahi-machi, Abeno-ku, Osaka, 545-8586 Japan; 3 Osaka City University Medical School Skills Simulation Center, 1-2-7, Asahi-machi, Abeno-ku, Osaka, 545-0051 Japan; 4 Department of Obstetrics and Gynecology, Osaka City University Graduate School of Medicine, 1-4-3, Asahi-machi, Abeno-ku, Osaka, 545-8585 Japan; 5 Department of Urology, Suita Municipal Hospital, 2-13-20, Katayama-cho, Suita-shi, Osaka, 564-0082 Japan

**Keywords:** Kidney transplantation, Pregnancy, In vitro fertilization, Rituximab, ABO-incompatible

## Abstract

**Background:**

Successful pregnancy outcomes after in vitro fertilization in kidney transplant recipients have been reported, but few cases of successful pregnancy after ABO-incompatible kidney transplantation have been described. Herein, we report on a successful pregnancy after in vitro fertilization in an ABO-incompatible kidney transplant recipient with rituximab, focusing on the changes in immunity.

**Case presentation:**

A 35-year-old woman with end-stage kidney disease caused by IgA nephropathy was referred for kidney transplantation and successfully underwent an ABO-incompatible living-donor kidney transplant using rituximab from her 66-year-old father at the age of 36. Because she and her husband desired childbearing, they received fertility treatments, and embryo cryopreservation was performed before transplantation. Two years after the transplant, she desired pregnancy. Although immunoglobulin levels such as IgG, IgA and IgM had recovered to almost normal range, the peripheral CD19^+^ cells and CD20^+^ cells remained depleted. At 6 months after conversion from mycophenolate mofetil to azathioprine, frozen embryo transfer was performed during the hormone replacement cycle.

At 37 weeks and 4 days gestation, a healthy baby girl weighing 2220 g was delivered by cesarean section for arrest of labor. There were no complications in both the recipient and her baby during the perinatal period. At 5 years after the transplant, the recipient has had no major complications including rejection or infection.

**Conclusions:**

It is possible for women receiving ABO-incompatible kidney transplantation with rituximab to successfully become pregnant and deliver a heathy baby after in vitro fertilization, if IgG levels recover to normal range despite depleted peripheral blood B cells.

## Background

Chronic kidney disease is often accompanied by sexual dysfunction and infertility in women patients as a consequence of kidney failure-related endocrine aberration. Even if they become pregnant, the incidence of spontaneous abortion, premature birth and intrauterine growth restriction is high [1, 2]. For these patients, kidney transplantation is an important option, as it significantly improves fertility rate and fetal and maternal outcomes [3]. In Japan, ABO-incompatible kidney transplantation (ABO-IKT) has been performed since the late 1980’s. ABO-IKT is immunologically a high-risk procedure because of antibody-mediated rejection due to anti-A/B antibodies. Few cases of successful pregnancy after ABO-IKT have been described [4–6], one of which was a pregnancy after in vitro fertilization (IVF) in an ABO-IKT recipient with rituximab [6]. Herein, we report on a case of successful pregnancy after IVF in an ABO-IKT recipient with rituximab, focusing on the changes in immunity during pregnancy.

## Case presentation

**Fig. 1:**
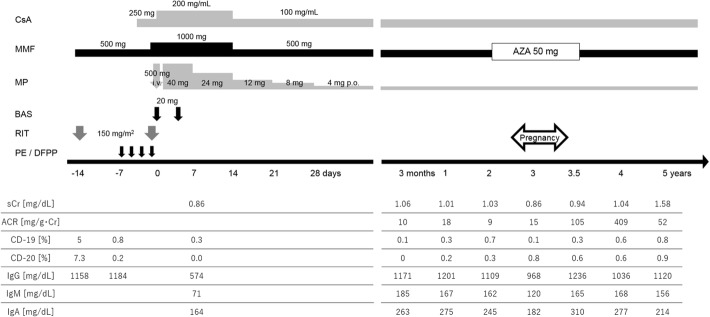
Immunosuppressive therapy, and changes in immunity and renal functionCsA; cyclosporine, MMF; mycophenolate mofetil, MP; methylprednisolone, BAS; basiliximab, RIT; rituximab, PE; plasma exchange, DFPP; double filtration plasmapheresis, AZA; azathioprine, sCr; serum creatinine, ACR; albumin to creatinine ratio

A 35-year-old woman, gravida 1, para 0, with end-stage kidney disease caused by IgA nephropathy was referred for kidney transplantation. Hemodialysis was initiated when she was 33 years old. She first became pregnant after starting hemodialysis and experienced spontaneous abortion at 5 months after initiation of hemodialysis. After experiencing spontaneous abortion, she received fertility treatments and tried in timed intercourse with fertility drugs. She decided to receive kidney transplantation in order to restore fertility. Embryo cryopreservation was performed considering her age before her first visit to our hospital, because she and her husband desired childbearing. She underwent an ABO-incompatible living-donor kidney transplant using rituximab from her 66-year-old father at the age of 36. Initial anti-A antibody titers were 1:128 (IgM) and 1:128 (IgG). Because she underwent two doses of rituximab infusion (150 mg/m^2^ on day 14 before and at transplantation) for B cell depletion and four courses of plasma exchange and double filtration plasmapheresis to remove antibodies, anti-A antibody titers were reduced to 1:8 (IgM) and 1:8 (IgG). She received maintenance immunosuppressive therapy including cyclosporine, mycophenolate mofetil and methylprednisolone after transplantation. The serum creatinine level increased from 1.3 to 1.6 mg/dl on the postoperative day 18. Two years after the transplant, because she had no rejection during the past year and had adequate and stable graft function with no acute infections as well as stable maintenance immunosuppression, she desired pregnancy. Although immunoglobulin levels such as IgG, IgA and IgM had recovered to almost normal range, the peripheral CD19^+^ cells and CD20^+^ cells remained depleted (Fig. 1). At 6 months after conversion from mycophenolate mofetil to azathioprine, frozen embryo transfer was performed during the hormone replacement cycle.

During pregnancy, the serum creatinine level was 0.8–1.0 mg/dl, and blood pressure was 120–130/70–80 mmHg. Although the cyclosporine trough level decreased to approximately 50 ng/ml after the 10th week of pregnancy, the dose of cyclosporine was not adjusted, because pregnancy seems to be a state of immunological tolerance associated with immunosuppressant activity of lymphocytes which creates tolerance to fetus [7]. The serum creatinine level elevated to 1.15 mg/dl at 36 weeks and 3 days gestation, and labor was induced at 37 weeks and 1 day gestation. At 37 weeks and 4 days gestation, a baby girl weighing 2220 g was delivered by cesarean section for arrest of labor, with Apgar scores of 8 and 9 at 1 and 5 min, respectively. The baby exhibited no malformation and was healthy. Serum IgG and IgM levels in the maternal cord blood were 912 and 16 mg/dL, respectively. The serum creatinine level improved to approximately 0.8–1.0 mg/dl after childbirth without treatment. Her blood pressure was stable until delivery but elevated to approximately 160/100 mmHg 4 days after childbirth, and she required antihypertensives for two weeks. She did not breastfeed her baby. Depletion of the peripheral blood CD19^+^ cells and CD20^+^ B cells due to administration of rituximab continued during pregnancy (Fig. 1). After pregnancy, anti-A antibody titers were 1:2 (IgM) and 1:2 (IgG). There were no complications in both the recipient and her baby during the perinatal period. At 5 years after the transplant, the recipient has had no major complications including rejection or infection, while the peripheral blood CD19^+^ cells and CD20^+^ B cells remain depleted due to administration of rituximab, and she is in good clinical condition with only mild renal insufficiency (serum creatinine 1.47 mg/dL) (Table 1). She said that she was completely happy with childbirth after kidney transplantation and is satisfied with childcare.

**Table 1:** Medical history timeline

Dates	Relevant Past Medical History
	Hemodialysis was initiated due to IgA nephropathy.
5 months after dialysis initiation	Her first pregnancy resulted in spontaneous abortion.
27 months after dialysis initiation	Embryo cryopreservation was performed.
8 months before transplantation	
Dates	Summary
28 months after dialysis initiation	The patient was referred for kidney transplantation.
7 months before transplantation	
35 months after dialysis initiation	ABO-incompatible living-donor kidney transplant using rituximab was performed.
25 months after transplantation	The patient was converted from mycophenolate mofetil to azathioprine.
15 months before pregnancy	
32 months after transplantation	Frozen embryo transfer was performed during the hormone replacement cycle.
8 months before pregnancy	
40 months after transplantation	She delivered a healthy baby but with a low birth weight.
3 years after childbirth	The patient is in good clinical condition with only mild renal insufficiency.

## Discussion and conclusions

Kidney transplantation is an important option for women with end-stage kidney disease contemplating pregnancy [8]. Pregnancy after kidney transplantation is proof of success, although it is not devoid of risks such as preterm birth, low birth weight and small for gestational age. ABO-IKT has become an established form of renal replacement therapy. Recently, Rao NN et al. reported a case of successful pregnancy after IVF in an ABO-IKT recipient receiving rituximab [6]. In this present case, we demonstrated a successful pregnancy after IVF in an ABO-IKT recipient with rituximab, focusing on the changes in immunity. Pregnancy in ABO-IKT recipients receiving rituximab may be achieved, if IgG levels recover to normal range despite depleted peripheral blood B cells.

In this case report, we evaluated the changes in immunity such as IgG, IgM, IgA and CD19^+^ and CD20^+^ B cells in the peripheral blood. Desensitizing with apheresis and rituximab-based immunosuppression decreased IgG levels and the number of CD19^+^ and CD20^+^ B cells in the peripheral blood at 7 days after transplantation. IgG returned to normal levels at 3 months after transplantation, but CD19^+^ and CD20^+^ B cell depletion was prolonged during pregnancy. It was previously reported that patients given rituximab may develop transient hypogammaglobulinemia and/or delayed B-cell reconstitution, and therefore should be routinely monitored for serum IgG, IgM and IgA as well as B-cell counts [9, 10]. Moreover, pregnant patients who developed transient hypogammaglobulinemia after rituximab should be monitored with neonatal serum IgG, and it might be better to avoid pregnancy after ABO-IKT with administration of rituximab until the patient recovers from hypogammaglobulinemia. We have experienced some patients with prolonged (over 3 years) peripheral B cell suppression after rituximab administration [11]. We routinely verified peripheral CD19^+^ and CD20^+^ B cell count after kidney transplantation, because this patient wanted to become pregnant.

Favorable outcomes of pregnancies after IVF are commonly reported, although IVF pregnancies are associated with an increased risk of preterm births, very low birth weight and small for gestational age compared with pregnancies after spontaneous conception due to the high proportion of multiple pregnancies [12]. There were 9 reports (16 pregnancies) published covering successful pregnancy outcomes after IVF in kidney transplant recipients [13–20]. Nine pregnancies were preterm, before 37 completed weeks of gestational age, and 4 pregnancies were twins. Previous reports showed that careful selection of women with kidney transplantation for IVF treatment may result in outcomes compatible to those in women who undergo kidney transplant and spontaneous conception [19]. In the present case, cesarean section was performed at 37 weeks and 4 days, and a healthy baby was delivered with a low birth weight (2220 g). This birthweight was very slightly lighter than the birthweight of the 10th centile at 37 weeks and 4 days gestation in the Japanese baby girl neonatal growth chart (2232 g) [21]. Although this finding may be a clinically relevant adverse outcome, it does not necessarily mean that IVF treatment should be avoided for women who had received ABO-IKT.

Although hyperprolactinemia in women with ESKD has been well documented, its mechanism is still not clear. In ESKD patients, serum luteinizing hormone and follicle-stimulating hormone concentrations increase while serum progesterone concentrations decrease. The increase in gonadotropin levels is attributable to the loss of negative feedback on hypothalamic and pituitary centers, and the luteinizing hormone surge is blocked. This abnormal hypothalamus-pituitary-ovarian axis leads to menstrual cycle irregularity, anovulation, decreased libido, and impaired fertility. It has been reported that at 2 to 3 weeks after kidney transplantation, there is a temporary improvement in hypogonadotropic hypogonadism, which is followed by normalization of hypothalamic-gonadal functions. The levels of circulating sex steroids remain suppressed and return to normal levels at 6 months after transplantation [22]. Moreover, although immunosuppressants have been reported to be teratogenic based on animal experiments, previous studies have confirmed safety of some agents in pregnancy. Women transplant patients receiving immunosuppressants whose safety has not been established must therefore be converted to those whose safety has been confirmed. Pregnancy can cause hyperfiltration, intrarenal vasodilation, and increased effective plasma flow without elevating intraglomerular pressure. It has also been reported that a renal allograft can adapt to the physiological changes of pregnancy in which creatinine clearance increases approximately 30% in the first trimester, slightly decreases in the second trimester and returns to pre-pregnancy levels during the third trimester. In addition, glomerular hyperfiltration during pregnancy is known to be transient and not accompanied by permanent renal impairment [23].

The optimal time to conception after kidney transplant remains controversial. Moreover, no reports have been made on the optimal time after ABO-IKT. In this case, we advised her to wait at least 2 years after ABO-IKT to ensure the safety of pregnancy, in reference to European best practice guidelines for kidney transplantation [24]. In pregnancies of kidney transplant recipients, major maternal complications are hypertension and pre-eclampsia, and major fetal complications are preterm delivery, low birth weight, and small for gestational age [7]. In this case, there were temporary hypertension, elevation of albuminuria, low birth weight, and small for gestational age, but no pre-eclampsia and preterm delivery. The albumin to creatinine ratio increased, 100–400 mg/g, during three months of pregnancy and a year after delivery (Fig. 1). There is a possibility that delivery contributed to the decline in graft function. However, transplant biopsy undertaken a year after delivery showed no significant pathological changes.

In conclusion, our case demonstrated that it was possible for women who received ABO-IKT with rituximab to successfully become pregnant and deliver a heathy baby after IVF, if IgG levels recover to normal range despite depleted peripheral B cells Further research is needed to establish the optimal time for conception and safety of IVF after ABO-IKT with rituximab.

## Data Availability

Not applicable.

## Abbreviations

ABO-IKT: ABO-Incompatible kidney transplantation
IVF: In vitro fertilization
